# Discrimination of Rice with Different Pretreatment Methods by Using a Voltammetric Electronic Tongue

**DOI:** 10.3390/s150717767

**Published:** 2015-07-22

**Authors:** Li Wang, Qunfeng Niu, Yanbo Hui, Huali Jin

**Affiliations:** 1 School of Electrical Engineering, Henan University of Technology, Zhengzhou 450007, China; E-Mails: niuqunfeng@haut.edu.cn (Q.N.); huiyb@haut.edu.cn (Y.H.); 2 School of Food Science and Engineering, Henan University of Technology, Zhengzhou 450007, China; E-Mail: jinhuali@haut.edu.cn

**Keywords:** rice, discrimination, variety prediction, voltammetric electronic tongue, cyclic voltammetry, fast Fourier transform, Discriminant Factorial Analysis, radial basis function neural network

## Abstract

In this study, an application of a voltammetric electronic tongue for discrimination and prediction of different varieties of rice was investigated. Different pretreatment methods were selected, which were subsequently used for the discrimination of different varieties of rice and prediction of unknown rice samples. To this aim, a voltammetric array of sensors based on metallic electrodes was used as the sensing part. The different samples were analyzed by cyclic voltammetry with two sample-pretreatment methods. Discriminant Factorial Analysis was used to visualize the different categories of rice samples; however, radial basis function (RBF) artificial neural network with leave-one-out cross-validation method was employed for prediction modeling. The collected signal data were first compressed employing fast Fourier transform (FFT) and then significant features were extracted from the voltammetric signals. The experimental results indicated that the sample solutions obtained by the non-crushed pretreatment method could efficiently meet the effect of discrimination and recognition. The satisfactory prediction results of voltammetric electronic tongue based on RBF artificial neural network were obtained with less than five-fold dilution of the sample solution. The main objective of this study was to develop primary research on the application of an electronic tongue system for the discrimination and prediction of solid foods and provide an objective assessment tool for the food industry.

## 1. Introduction

Rice plays a significantly important role in people’s daily life, therefore, detection of the rice quality has received progressively increasing attention. However, currently, the evaluation of rice quality lacks a uniform standard and the evaluation method depends on the test aim of the rice. Rice quality has attracted significant attention and it has become the most important target in rice improvement. Therefore, the quality of rice is evaluated in terms of its sensory quality, processing quality, eating quality, and nutritional quality. The assessment indexes of sensory quality of rice are mainly based on the color, appearance, smell, taste and other features which are identified by the examiner’s sense organs and practical experience. Rice processing quality mainly reflects the characteristics of rice processing. The main evaluation index includes milled rice rate and machining accuracy. The main assessment indexes of eating quality of rice include the gelatinization temperature, amylose content, and gel consistency. Evaluation of the nutritional quality of rice is mainly embodied in the detection of the content of rice starch, fat, protein, vitamins, and microelements which are beneficial to the human body. However, in some of these methods, the influence of the examiner’s subjective consciousness in the evaluation process is strong, and the validity and reliability of the corresponding evaluation results cannot be guaranteed. What’s more, pretreatment procedures, while highly desirable in order to obtain reliable evaluation results, are complex and time-consuming. With an increasing need for field rice harvest season and rice manufacture testing, it is necessary to study the discrimination and recognition of different varieties of rice and further to evaluate its quality control. Nowadays, modern analytical techniques are applied for the discrimination and identification of damaged rice plants. Furthermore, these techniques are also used for the discrimination of rice varieties based on volatile compounds released by the plant, and for rice detection by gas chromatography-mass spectrometry (GC-MC) or electronic noses [1,2,3,4,5,6].

Recently, electronic tongues have started to play an important role in the food industry as intelligent analytical systems. According to the IUPAC definition [7], an electronic tongue is “a multisensor system, which consists of a number of low-selective sensors and uses advanced mathematical procedures for signal processing based on pattern recognition (PARC) and/or multivariate data analysis artificial neural networks (ANNs), principal component analysis (PCA), *et al.*”. Therefore, an electronic tongue represents a new way to interpret chemical signals and provide qualitative and quantitative assessment of multispecies solutions. The two emerging analytical technologies of electronic tongues with electrochemical sensors and bioelectronic tongues involving biosensor arrays are studied in food applications [8,9,10].

When an electronic tongue is employed, there are two key parameters that determine the detection of information, *i.e.*, the modeling tool and the type of sensors used [11]. Voltammetric electronic tongues with voltammetric sensors are used extensively in electronic tongue systems. They have been applied for the qualitative/quantitative analysis of natural samples, wine, milk, tea, oil, or other liquid solutions [12,13,14,15,16,17,18,19,20,21,22,23,24]. An array of noble metal working electrodes has been used in voltammetric electronic tongues to analyze different foodstuffs, tea, juices, and milk [12,13,14,15,16]. Moreover, various materials such as polymers and epoxy-graphite have been used as coating membranes for sensor design. For example, Cetó *et al.*, developed a novel voltammetric electronic tongue containing modified epoxy-graphite electrodes for the qualitative and quantitative analysis of wine and beer [17,18,19,20,21,22,23,24].

Advanced data processing and pattern recognition methods are the fundamental part in any voltammetric electronic tongue system [11]. Among previously reported studies on qualitative modelling and various data processing methods implemented, PCA has been mostly used as a qualitative visualization tool; and partial least squares Discriminant Analysis (PLS-DA), Linear Discriminant Analysis (LDA), cluster algorithm (CA), Support Vector Machine (SVM), and ANNs have been employed as qualitative modeling [11,25,26,27,28,29,30,31,32] methods. However, Multiple Linear Regression (MLR), Principle Components Regression (PCR), PLS, or ANN are usually quantitative analysis tools [33,34,35,36,37,38,39]. Despite the significant advantages provided by the reported data processing methods, the high complexity of the obtained data matrix makes the model building very difficult for the use of voltammetric sensors, therefore, some feature extraction and preprocessing methods for data compression and reduction must be applied to reduce input data dimensionality, training time, and to obtain better model ability. The data preprocessing may be achieved by the use of PCA, fast Fourier transform (FFT), or discrete wavelet transform (DWT) [11,20,39].

The abovementioned discussion indicates that the voltammetric electronic tongue has several advantages and convenient features in the detection of liquid samples; however, the effect of taste detection in a solid food such as rice is unknown. Rice solutions which are dissolved in water contain certain water-soluble vitamins, inorganic salts, minerals, and rice bran oil containing a rich vitamin B and E composition. Different varieties of rice contain different quantitative water-soluble contents. Discrimination and recognition of the rice samples, with different quantities of this content, have seldom been evaluated using an electronic tongue taste sensor array. Consequently, it was necessary to develop an initial study to investigate whether the voltammetric array of sensors based on basic metal electrodes could detect these differences to output response signals of different samples under the excitation potential and then reflect the individual differences among sample solutions of different varieties of rice with proper data processing. Furthermore, in this study, an attempt was made to select a rice solution pretreatment method and to determine whether the rice needs to be crushed or not.

Thus, the main objective of this study was to construct a basis for the future use of a voltammetry-based electronic tongue, employing an array of metallic sensors for discrimination and prediction of different varieties of rice during rice harvest, storage, and processing. Moreover, a better and simpler rice sample pretreatment method was also proposed by comparing two such pretreatment methods. Specifically, an array of metallic working electrodes was applied as the sensing part. Cyclic voltammetry was set as excitation potential to obtain electrochemical response signals. The collected taste response signal data were first compressed by FFT. Discriminant Factorial Analysis (DFA) and radial basis function (RBF) artificial neural network were used as the discrimination and prediction modeling tools, respectively. Thus, this study provides a new perspective to the understanding of the discrimination and prediction of different varieties of rice by DFA and RBF artificial neural network by using an electronic tongue.

## 2. Materials and Methods

### 2.1. Materials

#### 2.1.1. Rice

A total set of 16 rice samples from different grain companies was analyzed. All rice samples considered in this study were commercially purchased. Four different varieties of rice, namely, JiaHe66, JiaHe218, XiuShui128, and XiuShui134 were selected and used as experimental samples. Four samples belonging to each variety were used for evaluation. The experimental samples are listed in Table 1.

**Table 1:** Experimental samples with different four varieties.

Brand	Rice Varieties	Working Electrode Array
JiaHe	JiaHe66	Pt
JiaHe	JiaHe218	Au
XiuShui	XiuShui134	Pd
XiuShui	XiuShui128	Ag

#### 2.1.2. Main Instruments and Materials

A total of four voltammetric sensors were selected with an array of metallic working electrodes, including platinum (Pt), silver (Ag), gold (Au), and palladium (Pd) electrodes (diameter 2 mm) according to preliminary exploratory experiments performed in our laboratory. A counterelectrode (Φ1 × 5 platinum column) and an Ag/AgCl reference electrode (diameter 6 mm) were selected to form the voltammetric measurement cell. Polishing materials included α-Al_2_O_3_ powders (1.5 μm, 0.5 μm, and 50 nm) and a polishing cloth (80 × 80 mm). All the electrodes and polishing materials were commercially purchased from Aidahengsheng Technology Co., Ltd. (Tianjing, China). The electrochemical workstation CS350 used to build the electronic tongue system was a commercial device purchased from Corrtest Instrument Co., Ltd. (Wuhan, China). The JA2003 electronic balance (0.001 g of division value and ±0.002 g of linear error) used for weighing the rice samples was supplied by the Precision Instruments Co., Ltd. (Shanghai, China).

### 2.2. Methods

The objective of this study was to investigate the ability of a metallic electrode-based electronic tongue to detect differences among different rice samples and provide discrimination and prediction results by using appropriate data processing methods. The other objective was to find a better and simple pretreatment method for the rice sample solutions prior to its practical application. Briefly, rice samples under test must be in solution. It was not determined whether rice need to be crushed or not when it was soaked in distilled water. Therefore, two types of rice sample solution were prepared by using crushed rice and non-crushed rice, respectively, and then subjected to DFA discrimination. In RBF neural network modeling, we also wanted to explore when the electronic tongue could not detect the information of rice sample solutions and give bad prediction effects with the increasing degree of dilution.

Therefore, a total set of 16 rice samples was prepared, respectively, with two pretreatment methods aiming to determine which method should be selected for further RBF neural network modeling and as final sample pretreatment method for field rice discrimination and prediction. The first pretreatment method involved the crushing of rice followed by its mixing with distilled water; however, the second method involved the direct mixing of rice, without any preprocessing, with distilled water.

#### 2.2.1. Preparation of the Crushed Rice Sample Solutions for Discrimination by DFA Model

The crushed rice sample solutions for discrimination were prepared prior to the measurement with the electronic tongue. First, an aliquot (50 g) from each rice sample was weighed and then placed in sequence into a pulverizer for crushing. Then, each crushed rice sample was placed in a mortar, for deep milling. Crushed and milled rice flour (20 g) was weighed and placed in a beaker according to the corresponding sample numbers. Subsequently, distilled water (100 mL) was mixed with the milled rice flour samples, respectively, stirred for one minute and the mixed solutions were filtered with a funnel. Finally, filtered liquid (80 mL) was taken as a final rice sample solution. Therefore, in sequence, the total set of 16 rice samples were pretreated according to the steps described above and then kept for the subsequent measurements by the electronic tongue system. In all the preparations of the crushed rice sample solutions, the pulverizer and the mortar must be cleaned with a small brush after each rice sample was crushed and deeply milled in order to maintain the purity of each rice sample.

#### 2.2.2. Preparation of the Non-Crushed rice Sample Solutions for Discrimination by DFA Model

Rice (20 g) from each variety of rice sample was measured and soaked in distilled water (100 mL) for 10 min, stirred at the first minute and the last minute. Then, the solutions were filtered using a funnel. Finally, filtered liquid (80 mL) was taken as final sample solution for the subsequent tests.

#### 2.2.3. Preparation of the Test Samples for RBF Neural Network Modeling

The test samples were prepared according to the following procedure: first, rice (20 g) from each rice sample was weighed, and placed in volumetric flasks marked with different sample number and variety. The volume of the volumetric flasks was 300 mL. Then, distilled water (150 mL) was added to these four volumetric flasks, respectively. Flasks were shaken for 2 min, and then allowed to stand for 10 min to provide sufficient time for the water-soluble substances to dissolve in water. Finally, the 16 mixture solutions were filtered through filter paper and volume of the filtration liquid was made up to 100 mL, respectively, and these solutions were used as the first group of rice sample solutions.

The second group of rice sample solutions was obtained by following a similar procedure. The next step was to dilute the test samples to four different concentrations: 0 times dilution, 5 times dilution, 10 times dilution, and 100 times dilution. First, 80 mL of the solution was taken from first group of rice sample solutions, respectively, and they were considered as the 0 times dilution test samples. Then, the 5 times dilution, 10 times dilution, and 100 times dilution of the test samples were obtained from the second group of rice sample solutions by the following procedure: separately 1, 10, and 20 mL of the solution was taken from the second group of rice sample solutions. Subsequently, they were diluted to 100 mL (corresponding to 100 times dilution of test samples, 10 times dilution, and 5 times dilution, respectively) and 80 mL of each diluted sample solution was taken out for further testing. Thus, four concentrations of each rice sample were obtained in total. Every rice sample solution was tested 5 times by cyclic voltammetry, thus 80 samples were to be tested at each concentration.

#### 2.2.4. Electronic Tongue

The voltammetric electronic tongue system was constructed with a voltammetric measurement cell, CS electrochemical workstation, and a PC. The voltammetric measurement cell was formed by a 21344-sensor voltammetric array, a reference Ag/AgCl electrode, and a platinum counter electrode. The counter electrode was placed at the same distance with different working electrodes around it, in order to reduce the interference caused by solution resistance. Taste electrochemical responses current data were obtained from cyclic voltammetry measurements using the CS electrochemical workstation. Cyclic voltammograms were obtained at room temperature (25 °C) on the PC.

The excitation signal of cyclic voltammetry is an isosceles triangle wave voltage. Starting from the initial potential *E_i_*, the potential varies linearly along a direction to the final potential *E_f_*, reversing immediately to go back to the initial potential. If there is no stop command, it will continue to repeat the abovementioned process. The general instrument potential scan rate can be from several mV to 1 V per second. Excitation potential was generated by the electrochemical workstation. Following are the cyclic voltammetry parameter settings in the system: initial potential: *E_i_* = −2 V; low potential: *E* = −2 V; high potential: *E* = +2 V; termination potential: *E_f_* = −2 V. The scan rate was 200 mV∙s^−1^; and the data sampling frequency was 100 Hz. Furthermore, to prevent the cumulative effect of impurities on the electrodes, an electrochemical cleaning procedure was performed between samples for 40 s (measure one time) in a beaker containing 80 mL distilled water.

The test samples were the total set of 16 rice sample solutions which were preprocessed by the two different pretreatment methods. To ensure the repeatability and stability of the response signals by the electronic tongue sensor, each solution was repeatedly measured and the measurement was repeated five times for each sample. Each test sample was measured by collecting 3986 points in each cyclic voltammetric measurement. Therefore, a dataset for 80 samples corresponding to 16 rice samples was obtained for analysis. The original data dimension of total samples formed a 5 (measuring times) × 16 (rice samples) = 80 lines, and 4 (four working sensors) × 3986 (collecting current data points of each sensor) column matrix.

#### 2.2.5. Data Processing

The main objective of this study was to assess rice sample pretreatment methods and the discrimination and prediction ability of the electronic tongue system. Furthermore, DFA was used to achieve the following two objectives: to assess rice sample pretreatment methods and to qualitatively assess discrimination ability of the electronic tongue system. RBF artificial neural network was used to evaluate the effect of the voltammetric electronic tongue prediction on unknown rice samples.

FFT was used for preprocessing voltammetric data which was done by using LabVIEW 8.5. DFA and RBF artificial neural network were performed for building discrimination and prediction models, respectively, by using MATLAB 7.1.

As explained before, the total data dimension of one measurement for the qualitative analysis of the rice samples was 4 (sensors) × 3986. Thus, significantly a large number of the voltammetric data was generated by using four voltammetric sensors. These data must be preprocessed before developing the models. This is due to the fact that if the complex input data were employed without preprocessing as model input, it would lead to several complications and difficulty in model building such as long training time, and complex weights or discriminant function computation, in particular, for the RBF artificial neural network. With this perspective, given the complexity of the input data, FFT was employed to compress the original data down to several Fourier coefficients in order to reduce the high raw data dimensionality and improve the models’ performance and to extract significant features from the voltammetric signals. FFT is a highly efficient Discrete Fourier Transform (DFT) algorithm. It is an efficient tool in digital signal processing which decomposes the large data sequence into different frequency coefficients. The appropriate selection criterion of the coefficients without loss of significant information was mainly determined by taking into consideration the two factors. First was fc which is defined as the ratio of the area intersected by raw current response curve and reconstruction signal curve to the total area under both the curves; fc reflects the signal reconstruction degree ranging from 0 to 1 depending on the similarity of the signals. Its value is 0 when the two signals have nothing in common, indicating the failure of the reconstruction of a raw signal. Its value increases with an increase in the reconstruction effect. When fc is 1, it represents the perfect reconstruction of the raw signal with the selected number of Fourier coefficients without any information loss. The second factor was compression ratio, which is defined as follows: (1 − number of Fourier coefficients/original current data) × 100%. The value of the compression ratio increases with increasing raw current response data compression degree [11,39]. Herein, the values highlighting the comparison of average fc with number of FFT coefficients for non-crushed rice samples are listed in Table 2. The values listed in Table 2 indicate that main characteristic of raw signal focused on some front coefficients with FFT decomposition. The raw signal reconstruction degree was more difficult to increase when achieving a certain value. In this manner, each raw data of 3986 points was compressed down to 16 coefficients and the compression ratio was up to 99.59% prior to the modeling.

**Table 2:** Comparison of average fc with number of FFT coefficients for non-crushed rice samples.

Number of Selected FFT Coefficients	fc			
	Pt	Ag	Au	Pd
FFT-4	0.8621	0.8004	0.7829	0.7863
FFT-8	0.8871	0.8106	0.8460	0.8368
FFT-16	0.9024	0.8126	0.9012	0.8482
FFT-32	0.9082	0.8183	0.9122	0.8565
FFT-64	0.9120	0.8192	0.9162	0.8587

In DFA discrimination modeling, the distance discriminant rule was used in the model building. The discrimination effect was expressed with a DFA map represented by discriminant functions as coordinates. The greater the value of the accumulated contribution rate of the first two discriminant functions, the more the original information can be represented only by the first two discriminant functions. The effect of DFA discrimination was judged in terms of Discrimination Index (DI) value. DFA made the space within similar samples as close as possible; however, the distance among different groups was larger. A DI with a value of 100 indicates a perfect discrimination among different groups.

A RBF neural network is a three-layer forward neural network with the radial basis function (Gaussian kernel function) as the activation function. The performance of a RBF neural network is mainly influenced by parameters including the number of the hidden layer nodes, radial basis function centre, and spread and weights between the hidden and the output layers. In this sense, it adopted the k-means clustering algorithm to determine the radial basis function centre of the hidden layer in training process. Spread value was determined as 1.5 times (named as overlap coefficient) the average distance between each cluster center. The number of the hidden layer nodes was first set with the former input sample numbers and then finally determined according to comparison of the success rate of prediction results. Output weights were adjusted with Least Square Method (LSM). In order to estimate the classification and prediction performance of the RBF neural network model, leave-one-out cross-validation was performed, where the original 79 samples were used for network training subset and one sample was left for network testing in turn. The obtained performance of the RBF neural network model was evaluated according to three different indicators: the classification success rate, specificity defined as percentage of objects from different classes correctly rejected by the model, and sensitivity defined as percentage of objects of each class identified by the classifier [21].

## 3. Results and Discussion

### 3.1. Voltammetry Characteristics of the Test Samples

**Figure 1:**
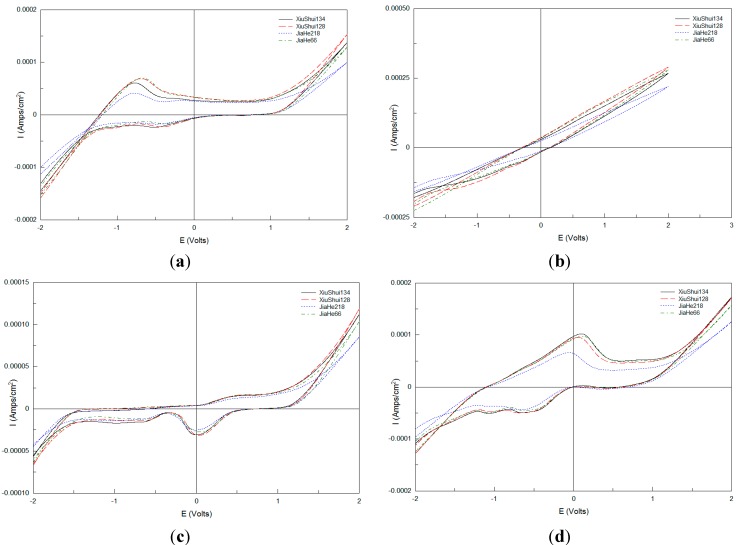
Voltammograms of four crushed rice sample solutions obtained using four electrodes. (**a**) Pt electrode; (**b**) Ag electrode; (**c**) Au electrode; (**d**) Pd electrode.

Figure 1 shows the voltammograms of four varieties of rice sample solution, obtained by the crushed rice pretreatment method, using four electrodes in the scanning potential range −2 to 2 V, at scan rate 200 mV∙s^−1^ under a linear change of potential. The upper half curve is the reduction wave which is called the cathodic branch, while the lower half part is the oxidation wave, known as the anode branch. Response current values reached their peak and trough under +2 V and −2 V.

**Figure 2:**
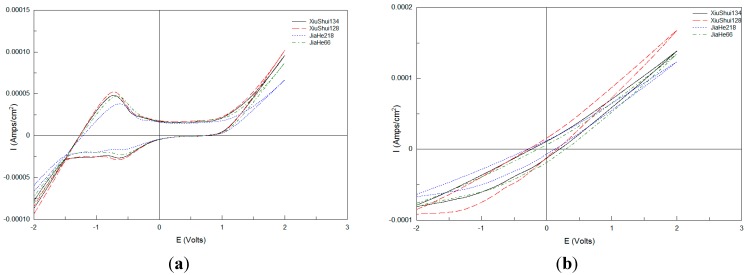

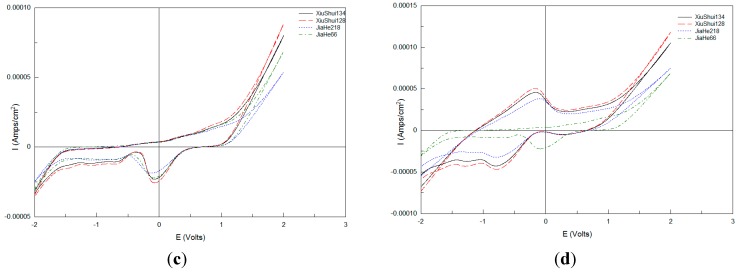
Voltammograms of four non-crushed rice sample solutions obtained using four electrodes. (**a**) Pt electrode; (**b**) Ag electrode; (**c**) Au electrode; (**d**) Pd electrode.

Figure 2 exhibits the voltammograms of four varieties of rice sample solution with the non-crushed rice pretreatment method obtained under the same condition. Figure 1 and Figure 2 show that both the four varieties of crushed and non-crushed rice sample solutions have a wide electrochemical window in which electrochemical reaction tests can be done and different cyclic voltammetry response curves under different electrodes were obtained with different varieties of rice tested.

### 3.2. Comparison of Current-Time Charts

**Figure 3:**
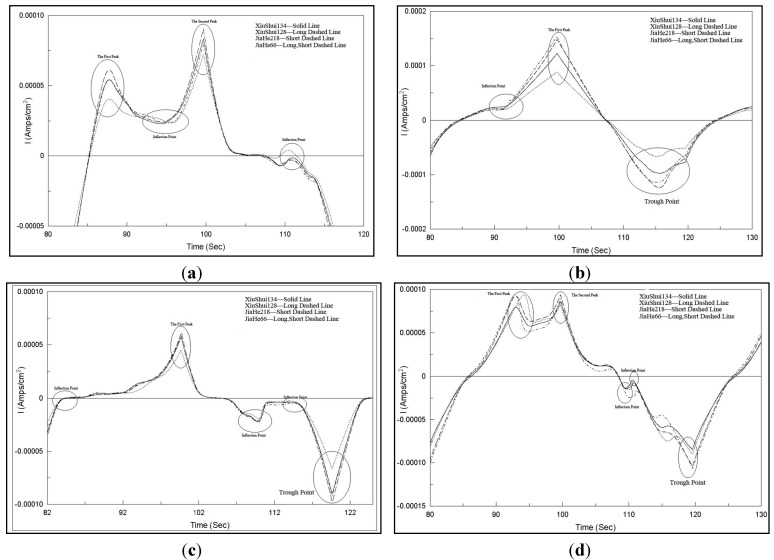
Current-time amplified charts of four working electrodes obtained with one time data collection for four crushed rice sample solutions. (**a**) Pt electrode; (**b**) Ag electrode; (**c**) Au electrode; (**d**) Pd electrode.

Current-time amplifying charts of all the four working electrodes obtained with one time data collection for four crushed rice sample solutions are shown in Figure 3, which exhibits similar response curves for different crushed rice sample solutions, except for different details, especially in the peaks, troughs, and the inflection point with the same working electrode. Obvious differences in the cyclic voltammetry response curves with different working electrodes are observed. The differences in details, especially in the peaks, troughs, and the inflection points are also circled in Figure 3. Different peaks, troughs, and the inflection points appeared by using different working electrodes. Five special data points are obtained with Pt electrodes (Figure 3a); however, three, five, and five special data points are obtained with Ag, Au, and Pd electrodes, respectively.

**Figure 4:**
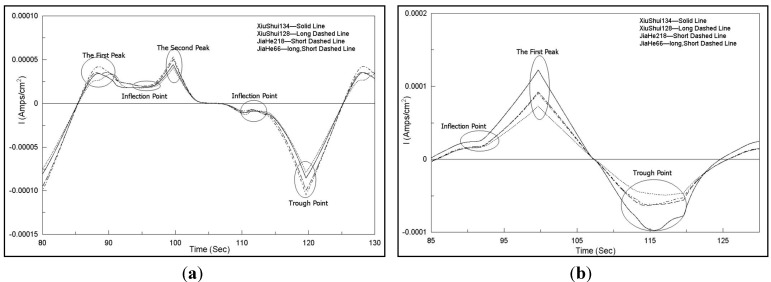

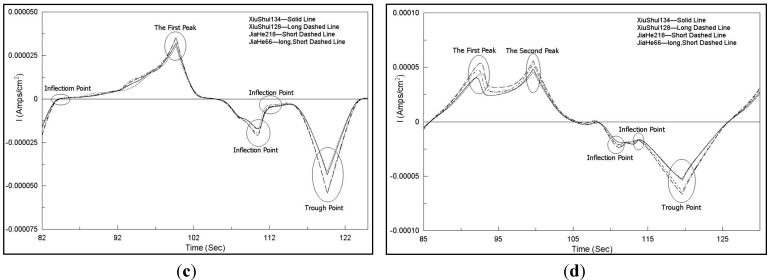
Current-time amplified charts of four working electrodes obtained with one time data collection for four non-crushed rice sample solutions. (**a**) Pt electrode; (**b**) Ag electrode; (**c**) Au electrode; (**d**) Pd electrode.

Similarly, the current-time charts for four non-crushed rice sample solutions are shown in Figure 4. Similar to the abovementioned descriptions, good response current-time characteristics were obtained with different sensors. Different peaks, troughs, and the inflection points also appeared by using different working electrodes. Similar special data points could be obtained in the same electrode voltammograms. However, comparison of Figure 3a and Figure 4a to Figure 3d and Figure 4d, corresponding to the same electrode voltammograms obtained after using different rice sample pretreatment methods, indicates that the total current response values with the crushed rice grain pretreatment method are higher than that with non-crushed pretreatment method. Consequently, more information could be obtained with crushed rice samples, which caused the sensor array of the electronic tongue to generate higher response current signals.

### 3.3. Discrimination of Different Rice Samples Using DFA

#### 3.3.1. Discrimination of Crushed Rice Sample Solutions

In order to compress the original data set and confirm the differentiated behavior, the original voltammetric responses data were compressed by using 16 FFT with the selected 64 FFT coefficients (each sensor response data corresponding to 16 FFT coefficients), and the obtained coefficients were analyzed by employing DFA.

Using the classical discriminant analysis method eigenvalues and accumulated contribution rate were obtained, which are listed in Table 3. The values listed in Table 3 indicate that the first discriminant function explains 86.8% of the original data information, the second function explains 12.9%, and the third function explains 0.4%. Notably, with just the first two discriminant functions, the accumulated contribution rate was up to 99.7%. Significantly large value indicated that most original data information could be represented by only these two new coordinates, leading to the determination of the differences in rice samples. Discrimination Index (DI) value was 96.

**Figure 5:**
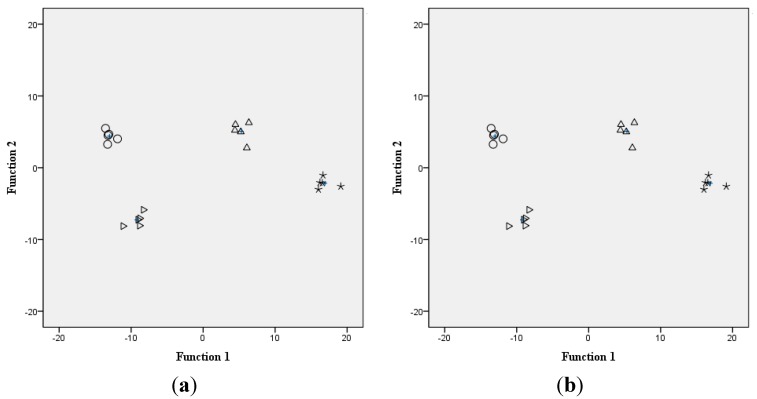
Discrimination of rice sample solutions by employing DFA analysis. (**a**) Crushed rice; (**b**) Non-crushed rice. (Ο) XiuShui134, (*) XiuShui128, (⊿) JiaHe218, and (Δ) JiaHe66, also the centroid of each class is plotted (+).

Figure 5a displays the discrimination effect plot obtained after DFA analysis of the four varieties of crushed rice sample solutions. Figure 5a clearly shows the separation of all the samples into four distinct clusters. The DFA plot shows that the sample solutions belonging to the same category are group in clusters around each group centroid, respectively. The same variety was nearby; however, the distance among the different groups was large. The above mentioned analysis showed that DFA could be a supervised method to distinguish different varieties of rice and the effect of discrimination was better.

**Table 3:** Classical discriminant analysis results for rice solutions.

Function	Crushed			Non-Crushed		
	Eigenvalue	Contribution	Correlation	Eigenvalue	Contribution	Correlation
D1	177.630	86.8%	0.997	1743.571	94.1%	1.000
D2	32.034	99.7%	0.985	13.982	98.5%	0.996
D3	0.041	100.0%	0.198	0.541	100.0%	0.593

#### 3.3.2. Discrimination of Non-Crushed Rice Sample Solutions

In order to compare the discrimination effect of the crushed rice sample solutions, the same voltammetric data preprocessed method using FFT and DFA analysis were used for the discrimination of non-crushed rice sample solutions.

Eigenvalues and accumulated contribution rate for the non-crushed rice sample solutions are also listed in Table 3. The values listed in Table 3 indicate that the first discriminant function explains 94.1% of the data information and the second function explains 4.4%. Thus, the total accumulated contribution rate of the first two discriminant functions is 98.5%, indicating that the first two discriminant functions can explain majority of the original data information without any significant loss in the information. Discrimination Index (DI) value is 98.

The discrimination plot of the rice sample solutions employing DFA analysis is shown in Figure 5b, revealing that different varieties of non-crushed rice sample solutions are distributed away from each other. The space within the same variety is less; however, the distance among the different groups was large, indicating good data repeatability and a clear distinction between different samples. Thus, the rice sample solutions with non-crushed pretreatment method can also be efficiently discriminated by DFA analysis.

The abovementioned analysis of the rice sample solutions through two different pretreatment methods showed that DFA could efficiently discriminate different rice samples obtained by both pretreatment methods, except that the current response values with non-crushed pretreatment were lower. The results of the crushed pretreatment method indicated that the four varieties of rice could be well distinguished with DI value of 96. Interestingly, the discrimination effect was also perfect for non-crushed pretreatment methods with DI value of 98. The pretreatment of crushed rice was more complex because it involved crushing, dissolving, centrifuging, and filtering prior to the test; therefore, non- crushed pretreatment methods could be considered as a better choice to perform discrimination and later recognition modeling of electronic tongue to simplify the rice pretreatment process and pretreatment time. The total DFA analysis results provided the basis for the optimization and selection of the rice pretreatment method.

### 3.4. Prediction of Different Unknown Samples of Rice Using RBF Neural Network

To further assess the ability of the electronic tongue for the identification of unknown rice samples, a RBF neural network prediction model was attempted. Good discrimination results were observed, which indicated that different varieties of rice could be distinguished by using the non-crushed sample solutions; therefore, further tests and analysis were performed on the four varieties of non-crushed rice samples. The test samples were obtained by the steps described in Section 2.2.3.

The recognition and prediction of the unknown variety of rice were modeled by using RBF neural network from the voltammetric responses data, previously compressed with FFT. The final structure of the RBF neural network was designed as 64-32-1 after a systematic study to optimize its topology. The number of input neurons was 64, and they were corresponding to the FFT coefficients of the four metallic sensors, and 16 separate coefficients for each sensor as before. Thirty two neurons were set in the hidden layer and one neuron in the output layer. The output values 1, 2, 3, and 4 correspond to “JiaHe218”, “JiaHe66”, “XiuShui128”, and “XiuShui134”, respectively.

The recognition and prediction effect of RBF neural network with leave-one-out cross-validation approach was studied. The recognition and prediction results are listed in Table 4. Rows indicated expected rice class and columns predicted ones. All the varieties of rice samples with four concentration gradients were measured and predicted. The values listed in Table 4 indicate that at 0 dilution, the recognition success rate of the four types of rice samples reached 95% of accuracy. As aforementioned, the obtained recognition efficiency of the RBF neural network also evaluated specificity and sensitivity. The value of specificity, averaged for the four classes considered was 98.3% and that of sensitivity was 95%. When recognizing rice samples with 5 times dilution, the correct recognition rate decreased significantly down to 85%, and when diluted 10 times and 100 times, the prediction accuracy of rice samples decreased down to 45% and 7.5%, respectively. The value of specificity also dropped from 95% to 81.7% and finally to 69.2% corresponding to rice sample solutions with dilution of 5 times, 10 times, and 100 times, respectively. Moreover, sensitivity dropped from 85% to 45% and finally to 7.5%. Thus, consequently, when the sample was diluted 100 times, the RBF neural network prediction model was less effective, it could correctly recognize only six out of a total 80 samples.

**Table 4:** Prediction results of RBF neural network using leave-one-out cross validation approach.

Dilution Times	Expected	Predicted						Sensitivity
		XiuShui134	XiuShui128	JiaHe218	JiaHe66	Accuracy	Specificity	
0	XiuShui134	20	0	0	0	95%	98.3%	95%
	XiuShui128	0	18	0	2			
	JiaHe218	1	0	19	0			
	JiaHe66	0	1	0	19			
5	XiuShui134	18	0	2	0	85%	95%	85%
	XiuShui128	1	16	0	3			
	JiaHe218	2	0	17	1			
	JiaHe66	0	2	1	17			
10	XiuShui134	10	3	7	0	45%	81.7%	45%
	XiuShui128	0	7	6	7			
	JiaHe218	6	0	10	4			
	JiaHe66	0	8	3	9			
100	XiuShui134	3	4	8	5	7.5%	69.2%	7.5%
	XiuShui128	4	1	5	10			
	JiaHe218	11	3	1	5			
	JiaHe66	3	12	4	1			

In all, the RBF neural network could be used effectively to distinguish different varieties of rice; however, at different concentrations, the ability of RBF neural network to identify rice samples was markedly different. When the sample dilution ratio was more than 10 times, RBF exhibited worse rice variety identification performance.

Finally, in order to confirm the effectiveness of the FFT data preprocessing or compressing method, other original voltammetric data preprocessing methods were attempted to compare the performance of the prediction model with different preprocessing methods. As stated in Section 3.2, different rice sample solutions exhibited different details especially in the peaks, troughs, and the inflection point circled in Figure 3 and Figure 4. The ability to recognize the rice variety using only these data details was observed. Response current peak value, the inflection point value, and trough value of each sensor for all 80 samples at each concentration were selected as the eigenvalues for model input. Concretely, five eigenvalues were extracted from Pt electrode, Au electrode, and Pd electrode, respectively. However, three eigenvalues were extracted from Ag electrode.

Therefore, the structure of the RBF neural network was designed as 18-13-1 after model optimization. The number of input neurons was 18, and they corresponded to the eigenvalues of the four metallic sensors, separately number 5, 5, 5, and 3 for each sensor, 13 neurons in the hidden layer and one neuron in the output layer. Consequently, the prediction accuracy was demoted to 91.25%, 77.5%, 40%, and 3.75% respectively corresponding to rice sample solutions with different dilution. Therefore, it was confirmed that in contrast to the preprocessing method of feature value extraction, the best RBF neural network performance was obtained by using FFT.

## 4. Conclusions

A voltammetric electronic tongue, based on the combination of metallic sensors, was researched in order to create a suitable tool for discrimination and prediction of different varieties of rice. The sensor array coupled with data compression method, statistical method, and pattern recognition method, namely, FFT, DFA, and RBF artificial neural network, respectively, were employed to discriminate and predict different types of rice. DFA was established for selecting the rice pretreatment methods and discrimination of different varieties. A RBF neural network with leave-one-out cross validation approach realized recognition and prediction. According to the RBF neural network model, 95% of rice sample solutions with no dilution were correctly recognized. The results of this study showed that the voltammetric electronic tongue was a useful tool for qualitative analysis of rice. This study undertook a preliminary exploration of taste assessment in solid food using voltammetric electronic tongue technology. However, the variety and quantity of samples were not sufficient for further research and the application effect of experimental results needs further study and in-depth discussion of practical applications. Further, different data preprocessing methods and more modeling are necessary to confirm the validity of this system for qualitative analysis of rice. Undeniably, a lot more systematic explorations are demanded for the detection of specific rice gustatory or non-volatile substances information by employing the electronic tongue.

## References

[1] D’Alessandro M., Turlings T.C.J. (2006). Advances and challenges in the identification of volatiles that mediate interactions among plants and arthropods. Analyst.

[2] Wang X., Zhou G.X., Xiang C.Y., Du M.H., Cheng J.A., Liu S.S. (2008). Beta-glucosidase treatment and infestation by the rice brown planthopper Nilaparvata lugens elicit similar signaling pathways in rice plants. Chin. Sci. Bull..

[3] Shao X.L., Zhang L.Y., Song W., Min G., Ju X.R. (2014). Rapid detection method for stored Indica rice by electronic nose. J. Chin. Cereals Oils Ass..

[4] Xu S., Zhou Z.Y., Lu H.Z., Luo X.W., Lan Y.B., Zhang Y., Li Y.F. (2014). Estimation of the age and amount of brown rice plant hoppers based on bionic electronic nose use. Sensors.

[5] Zhou B., Wang J. (2011). Use of electronic nose technology for identifying rice infestation by Nilaparvata lugens. Sens. Actuators B Chem..

[6] Zhou B., Wang J. (2011). Discrimination of different types damage of rice plants by electronic nose. Biosyst. Eng..

[7] Vlasov Y., Legin A., Rudnitskaya A., Di Natale C., D’Amico A. (2005). Nonspecific sensorarrays (electronic tongue) for chemical analysis of liquids (IUPAC Technical Report). Pure Appl. Chem..

[8] Ha D., Sun Q.Y., Su K.Q., Wan H., Li H.B., Xu N., Sun F., Zhuang L.J., Hu N., Wang P. (2015). Recent achievements in electronic tongue and bioelectronic tongue astaste sensors. Sens. Actuators B Chem..

[9] Gutés A., Céspedes F., Alegret S., del Valle M. (2005). Determination of phenolic compounds by a polyphenol oxidase amperometric biosensor and artificial neural network analysis. Biosens. Bioelectron..

[10] Gutiérrez M., Alegre S., del Valle M. (2008). Bioelectronic tongue for the simultaneous determination of urea, creatinine and alkaline ions in clinical samples. Biosen. Bioelectron..

[11] Cetó X., Céspedes F., del Valle M. (2013). Comparison of methods for the processing of voltammetric electronic tongues data. Microchim. Acta.

[12] Winquist F., Wide P., Lundström I. (1997). An electronic tongue based on voltam-metry. Anal. Chim. Acta.

[13] Winquist F., Krantz-Rülcker C., Wide P., Lundström I. (1998). Monitoring of freshness of milk by an electronic tongue on the basis of voltammetry. Meas. Sci. Technol..

[14] Winquist F., Holmin S., Krantz-Rülcker C., Wide P., Lundström I. (2000). A hybridelectronic tongue. Anal. Chim. Acta.

[15] Ivarsson P., Holmin S., Höjer N.E., Krantz-Rülcker C., Winquist F. (2001). Discrimination of tea by means of a voltammetric electronic tongue and different applied waveforms. Sens. Actuators B Chem..

[16] Winquist F., Lundström I., Wide P. (1999). The combination of an electronic tongueand an electronic nose. Sens. Actuators B Chem..

[17] Riul A., Malmegrim R.R., Fonseca F.J., Mattoso L.H.C. (2003). An artificial taste sensor based on conducting polymers. Biosens. Bioelectron..

[18] Gutiérrez J.M., Moreno-Barón L., Pividori M.I., Alegret S., del Valle M. (2010). A voltammetric electronic tongue made of modified epoxy-graphite electrodes for the qualitative analysis of wine. Microchim. Acta.

[19] Cetó X., Gutiérrez J.M., Moreno-Barón L., Alegret S., del Valle M. (2010). Voltammetric electronic tongue in the analysis of cava wines. Electroanalysis.

[20] Cetó X., Gutiérrez J.M. (2012). Determination of total polyphenol index in wines employing a voltammetric electronic tongue. Anal. Chim. Acta.

[21] Gutiérrez J.M., Amari A., Bouchikhi B., Mimendia A., Cetó X., Del Valle M. (2013). Hybrid electronic tongue based on multisensor data fusion for discrimination of beers. Sens. Actuators B Chem..

[22] Arrieta Á.A., Rodríguez-Méndez M.L., de Saja J.A., Blanco C.A., Nimubona D. (2010). Prediction of bitterness and alcoholic strength in beer using an electronictongue. Food Chem..

[23] Parra V., Arrieta Á.A., Fernández-Escudero J.A., Íniguez M., de Saja J.A., Rodríguez-Méndez M.L. (2006). Monitoring of the ageing of red wines in oak barrels bymeans of an hybrid electronic tongue. Anal. Chim. Acta.

[24] Dominguez R.B., Moreno-Baron L., Munoz R. (2014). Voltammetric electronic tongue and support vector machines for identification of selected features in Mexican coffee. Sensors.

[25] Men H., Zhang C.W., Zhang P.Y., Gao H.H. (2013). Application of electronic tongue in edible oil detection with cluster algorithm based on artificial fish swarm improvement. Adv. J Food Sci. Technol..

[26] Winquist F., Olsson J., Eriksson M. (2011). Multicomponent analysis of drinking water by a voltammetric electronic tongue. Anal. Chim. Acta.

[27] Tiwari K., Tudu B., Bandyopadhyay R., Chatterjee A. (2013). Identification of monofloral honey using voltammetric electronic tongue. J. Food Eng..

[28] Wu C.Y., Wang J., Wei Z.B. (2010). Prediction of apparent viscosity of milk with different volume fraction using electronic tongue. Trans. CSAE.

[29] Mottram T., Rudnitskaya A., Legin A., Fitzpatrick J.L., Eckersall P.D. (2007). Evaluation of a novel chemical sensor system to detect clinical mastitis in bovine milk. Biosens. Bioelectron..

[30] Wu R.M., Zhao J.W., Chen Q.S. (2011). Quality assessment of green tea taste by using electronic tongue. Trans. CSAE.

[31] Wei Z.B., Wang J., Ye L.S. (2011). Classification and prediction of rice wines with different marked ages by using a voltammetric electronic tongue. Biosens. Bioelectron..

[32] Han J., Huang L.J., Gu Z. (2008). Evaluation of fish quality and freshness based on the electronic tongue. Trans. CSAE.

[33] Rodriguez-Mendez M.L., Apetrei C. (2008). Evaluation of the polyphenolic content of extra virgin olive oils using an array of voltammetric sensors. Electrochim. Acta.

[34] Carpani I., Conti P., Lanteri S., Legnani P.P., Leoni E., Tonelli D. (2008). Direct quantification of test bacteria in synthetic water-polluted samples by square wave voltammetry and chemometric methods. Biosens. Bioelectron..

[35] Ghosh A., Tamuly P., Bhattacharyya N. (2012). Estimation of theaflavin content in black tea using electronic tongue. J. Food Eng..

[36] Ariza-Avidada M., Cuellar M.P., Salinas-Castillo A. (2013). Feasibility of the use of disposable optical tongue based on neural networks for heavy metal identification and determination. Anal. Chim. Acta.

[37] Valdés-Ramírez G., Gutiérrez M., del Vall M., Ramírez-Silva M.T., Fournier D., Marty J.L. (2009). Automated resolution of dichlorvos and methylparaoxon pesticide mixtures employing a Flow Injection system with an inhibition electronic tongue. Biosens. Bioelectron..

[38] Newman J., Harbourne N., ORiordan D., Jacquier J.C., O’Sullivan M. (2014). Comparison of a trained sensory panel and an electronic tongue in the assessment of bitter dairy protein hydrolysates. J. Food Eng..

[39] Cetó X., Céspedes F., del Valle M. (2015). Instrumental measurement of wine sensory descriptors using a voltammetric electronic tongue. Sens. Actuators B Chem..

